# Characterization of Insertion Sequence IS*Sau2* in the Human and Livestock-Associated *Staphylococcus aureus*

**DOI:** 10.1371/journal.pone.0127183

**Published:** 2015-05-15

**Authors:** Liangliang Wang, Huping Xue, Longping Li, Xin Zhao

**Affiliations:** 1 College of Animal Science and Technology, Northwest A&F University, Yangling, Shaanxi Province, People’s Republic of China; 2 Department of Animal Science, McGill University, Quebec, Canada; Ross University School of Veterinary Medicine, SAINT KITTS AND NEVIS

## Abstract

Mobile genetic elements play important roles in evolution and diversification of bacterial genomes. IS*Sau2* is 1660bp in length with terminal 5’-TG and CA-3’ dinucleotides and has two overlapping reading frames *orfA* and *orfB*. It has been found in a wide range of *S*. *aureus*, such as HA-MRSA252, LGA251, MRSA S0385 and ED133. To determine distribution of IS*Sau2*, 164 *S*. *aureus* isolates from milk samples of mastitic cows from our laboratory and all the *S*. *aureus* strains from the National Center for Biotechnology Information (NCBI) database were screened for the presence of IS*Sau2*. Next, in order to explore a potential relationship among *S*. *aureus* IS*Sau2*-containing strains and isolates, a relationship among 10 IS*Sau2*-positive *S*. *aureus* isolates and 27 IS*Sau2*-positive *S*. *aureus* strains was investigated by a phylogenetic analysis. These IS*Sau2* isolates and strains could be classified into four groups (A, B, C and D). The strains or isolates in Group D were all isolated from mammary glands, suggesting tissue specificity. All strains in Group B had an identical IS*Sau2* derivative, termed IS*Sau2*_1628_, with 32bp deletion at the 3’ terminus. IS*Sau2*_1628_ in strain ST398 from Group B was closely related to IS*Sau2* in strain LGA251 from Group D.

## Introduction

Insertion sequences (ISs) are the smallest autonomously mobile genetic elements, generally 700–2500 base pairs (bp) in size, and are widely distributed in both eukaryotic and bacterial genomes. ISs play an important role in genome evolution by gene inactivation, genome rearrangement, and genome reduction as well as activation or inactivation of neighboring gene expression [1]. They are abundant and affect the genomic plasticity and pathogenic potential of *S*. *aureus* [2]. *S*. *aureus* is both a commensal organism and a pathogen in humans. The anterior nares are the main ecological niche for *S*. *aureus* [3]. However, numerous other sites may be colonized, including the axillae, groin, and gastrointestinal tract [3]. Colonization provides a reservoir from which bacteria can be introduced when host defenses are breached, whether by shaving, aspiration, insertion of an indwelling catheter, or surgery, causing a number of invasive [3]. *S*. *aureus* also colonizes and affects a range of other animals including cows, pigs and goats [4].

Among over twenty IS families [1], the IS*3* family is a large group found in both Gram-negative and Gram-positive bacterial species [5]. The IS*3* family can be further divided into six subgroups (IS*150*, IS*407*, IS*51*, IS*3*, IS*2* and IS*911*). Most IS*3* family members share a common sequence organization in gene products and structural features, including the terminal dinucleotide 5'-TG-----CA-3', a small upstream open reading frame *(orfA)* and a longer downstream open reading frame *(orfB)*. The OrfAB transposase is a fusion protein produced from both open reading frames by programmed -1 translational frameshifting [6], with the sequence motif A_6_G as the frameshifting region [7,8]. OrfA, the deduced gene product from different IS*3*-like elements, typically contains a helix-turn-helix (HTH) motif that is crucial to direct the specific binding of the transposase to terminal inverted repeats [9]. Within OrfB, there is a conserved amino acid motif DDE which shares a strong homology with the catalytic site of retroviral integrases [10,11]. IS*Sau2* has similar gene products and structural features like most IS*3* family members and is classified into the IS*150* group in the ISfinder database.

Previously we found IS*Sau2* in the A region of *S*. *aureus* isolate E48 sdrC gene, which was isolated from a bovine mastitic cow in Canada [12]. It was also found in the literature in a wide range of *S*. *aureus*, such as HA-MRSA252 [13], LGA251 [14], MRSA S0385 [15] and ED133 [16]. The copy number and insertion sites of IS*Sau2* have been studied by whole genome sequencing of these strains. There were five copies in MRSA252, three in LGA251, one in MRSA S0385 and seven in ED133. Bioinformatics analyses of IS*Sau2* insertion sites in various *S*. *aureus* genomes did not reveal nucleotide sequence specificity for target sites [17]. Additionally, the copy number of IS*Sau2* was different in the three major clades (the clonal types were typically ST30*spa43* for clade 1, ST30*spa19* for clade 2, and ST36*spa16* or ST30*spa33* for clade 3) of *S*. *aureus* clonal complex (CC) 30 [17]. Whether other *S*. *aureus* isolates and strains also contain IS*Sau2* is worthy to be investigated. In addition, the relationship among the isolates and strains containing IS*Sau2* deserves exploration.

The aim of this study was to determine distribution of IS*Sau2* and to explore a potential relationship among *S*. *aureus* strains and isolates containing IS*Sau2*.

## Material and Methods

### PCR screening of bovine mastitis-associated *S*. *aureus* isolates for IS*Sau2* element

Genomic DNA was purified from 164 *S*. *aureus* isolates from milk samples of mastitic cows from our laboratory [18], using a Genomic DNA purification kit (Tiandz, Beijing, China). The presence of IS*Sau2* was determined using specific primer pairs (IS-F and IS-R, Table 1) and the size of the PCR product was 1660bp. The PCR conditions were as follows: denaturation at 95°C for 5 min, annealing at 58°C for 1 min, extension at 72°C for 2 min and final extension at 72°C for 7 min. The copy number of IS*Sau2* in each isolate was determined by real-time qPCR.

**Table 1:** List of the primers and probes used in this study.

	Primers Sequences
IS-F	TGAAATGCTCCCTTCAAAGTAGACA
IS-R	TGAACTGCACCCAGTCTAGTAGACAATT
SP1	ATTCGATTGCGAGAGCTTGGGTTG
SP2	GTAAGTAGTGCTTCACTACCATGGG
SP3	TCGATGAAGTATTGACACGACACGC

### Sequence analysis

IS*Sau2* DNA segments from IS*Sau2*-positive *S*. *aureus* isolates were cloned into pEASY-T5 and sequenced by Genscript (Nanjing, China). The BLAST software from NCBI (blastn) was used to determine the presence of IS*Sau2* in genomes of all published *S*. *aureus* using IS*Sau2* from the E48 isolate as the query sequence and Reference genomic sequences (refseq_genomic) as the database with default algorithm parameters (the database was accessed on May 12, 2014). For bioinformatics analysis, IS*Sau2* sequences from different *S*. *aureus* strains or isolates were aligned using the ClustalX program [19] and phylogenetic trees were then constructed employing the MEGA 5.2 program on a neighbor-joining algorithm and a maximum likelihood method [20]. The two methods produced similar results.

### Genomic walking

Genomic DNA was purified from nine IS*Sau2*-positive *S*. *aureus* isolates and E48 using a Genomic DNA purification kit (Genomic Walking Kit, Takara Biotechnology, Dalian, China). Three specific primers (SP1, SP2 and SP3 in Table 1) were designed from the known sequence of IS*Sau2* using the Primer 5 program. A three step PCR assay was performed according to manufacturer’s instructions.

## Results

### Distribution and copy numbers of IS*Sau2* in *S*. *aureus*

To determine the presence of IS*Sau2* in *S*. *aureus*, a PCR to amplify the transposase region (1565bp) was carried out using the genomes of 164 bovine mastitis-associated *S*. *aureus* isolates as templates. According to the PCR results, 5.49% of the *S*. *aureus* isolates (9 out of 164) were confirmed to contain IS*Sau2* (data not shown). A nucleotide BLAST (BLASTn) of IS*Sau2* was also performed, utilizing the Reference genomic sequences (http://www.ncbi.nlm.nih.gov/refseq/) database as templates. Based on the blast results (E-value is 0.0), 27 *S*. *aureus* strains were found to contain the IS*Sau2* insertion sequences.

The copy numbers of IS*Sau2* in *S*. *aureus* isolates were determined by Real-time qPCR. There are less than three copies in all the *S*. *aureus* isolates containing IS*Sau2* (Table 2). On the basis of the whole genome sequences, the copy numbers of IS*Sau2* in 27 published IS*Sau2*-positive *S*. *aureus* strains were also determined, ranging from 1 to 7 (Table 2).

**Table 2:** IS*Sau2*-positive *S*. *aureus* strains and isolates evaluated in this study.

Strain	Location	Year	MLST	CC	Number of IS*Sau2*	Number of IS*Sau2* sequence type	SCCmec	Host & Disease	Accession
MRSA252	Oxford, UK	1997	ST36	CC30	5	3	HA-MRSA	Human with Septicemia	NC_002952.2
MN8	Korea	1980	ST30	CC30	3	1	MSSA	Human	CM000952.1
M1256	ND	ND	ST30	CC30	2	1	ND	Human	NZ_KB822114.1
M0513	ND	ND	ST36	CC30	5	1	MRSAII	Human	NZ_KB821413.1
EMRSA-16	UK	1998	ST36	CC30	1	1	MRSAII	Human with Chest infections, Septicemia, Endocarditis;	NZ_GG770521.1
LCT-SA112	China	ND	ST243	CC30	1	1	ND	Human	NZ_JH691956.1
TCH60	TX, USA	-	ST30	CC30	2	2	CA-MRSA	Human	NC_017342.1
M0408	ND	ND	ST431	CC30	5	1	MRSA	Human	NZ_KB821331.1
M809	Australia	1961	ST30	CC30	1	1	MRSA	Human	NZ_GG749305.1
C101	UK	1997	ST30	CC30	1	1	MSSA	Human	NZ_GG730124.1
55/2053	UK	1955	ST30	CC30	3	1	MSSA	Human	NC_022113.1
HO_5096_0412	Suffolk, UK	2005	ST22	CC22	1	1	EMRSA-15	Human with Lethal neonatal infections	NC_017763.1
M1228	ND	ND	ST22	CC22	1	1	MRSA	Human	NZ_KB822377.1
21310	ND	ND	ST22	CC22	1	1	MRSA	Human	NZ_AFNP00000000.1
S0385	Netherlands	2006	ST398	CC398	1	1	CA-MRSA	Human with Endocarditis; Also infect swine and calves	NC_017333.1
DR10	Dominican	2007	ST398	CC398	1	1	MSSA	Human with Abscess	NZ_AIDT00000000.1
08BA02176	Canada	2008	ST398	CC398	1	1	LA-MRSA	Human with Postoperative infection	NC_018608.1
S1	France	2008	ST398	CC398	1	1	LA-MRSA	Bovine	NZ_AUPS00000000.1
S94	France	2009	ST398	CC398	1	1	CA-MSSA	Human with Bloodstream infections	NZ_AUPW00000000.1
S123	Dutch	2010	ST398	CC398	1	1	LA-MSSA	Swine	NZ_AUPU00000000.1
S130	Netherlands	2010	ST398	CC398	1	1	LA-MRSA	Swine	NZ_AUPT00000000.1
S100	France	2010	ST398	CC398	1	1	HA-MSSA	Human with Bloodstream infections	NZ_AUPV00000000.1
21331	ND	ND	ST398	CC398	1	1	MSSA	Human with Septicemia, Pneumonia	NZ_AGTV01000007.1
112808A	Switzerland	2008	ST398	CC398	1	1	MRSA	Bovine Mastitis	NZ_AHZK00000000.1
71193	NY, USA	2004	ST398	CC398	1	1	MSSA	Human	NC_017673.1
LGA251	UK	2007	ST425	-	3	1	MSSA	Bovine Mastitis	NC_017349.1
ED133	France	1997	ST133	CC133	7	2	MSSA	Ovine Mastitis	NC_017337.1
E48	Canada	2011	ND	-	1	-	MSSA	Bovine Mastitis	-
5–14	China	2012	ST2683	-	2	-	MSSA	Bovine Mastitis	-
5–16	China	2012	ST2683	-	1	-	MSSA	Bovine Mastitis	-
8–5	China	2012	ST2683	-	2	-	MSSA	Bovine Mastitis	-
8–6	China	2012	ST2683	-	1	-	MSSA	Bovine Mastitis	-
8–9	China	2012	ST2683	-	2	-	MSSA	Bovine Mastitis	-
8–19	China	2012	ST2683	-	1	-	MSSA	Bovine Mastitis	-
9–17	China	2012	ST2683	-	2	-	MSSA	Bovine Mastitis	-
9–18	China	2012	ST2683	-	1	-	MSSA	Bovine Mastitis	-
9–19	China	2012	ST2683	-	2	-	MSSA	Bovine Mastitis	-

“Number of IS*Sau2*”: the copy number of IS*Sau2* in the according strain or isolate.

“Number of IS*Sau2* sequence type”: the number of IS*Sau2* sequence type in one *S*. *aureus* strain or isolate.

“MRSA”: Methicillin-resistant *Staphylococcus aureus*.

“HA-MRSA”: Healthcare-Associated Methicillin Resistant *Staphylococcus aureus*.

“CA-MRSA”: Community-associated methicillin-resistant *Staphylococcus aureus*.

“LA-MRSA”: Livestock-associated methicillin-resistant *Staphylococcus aureus*.

“MSSA”: Methicillin Susceptible *Staphylococcus aureus*.

### Sequence diversity of IS*Sau2*

**Fig 1:**
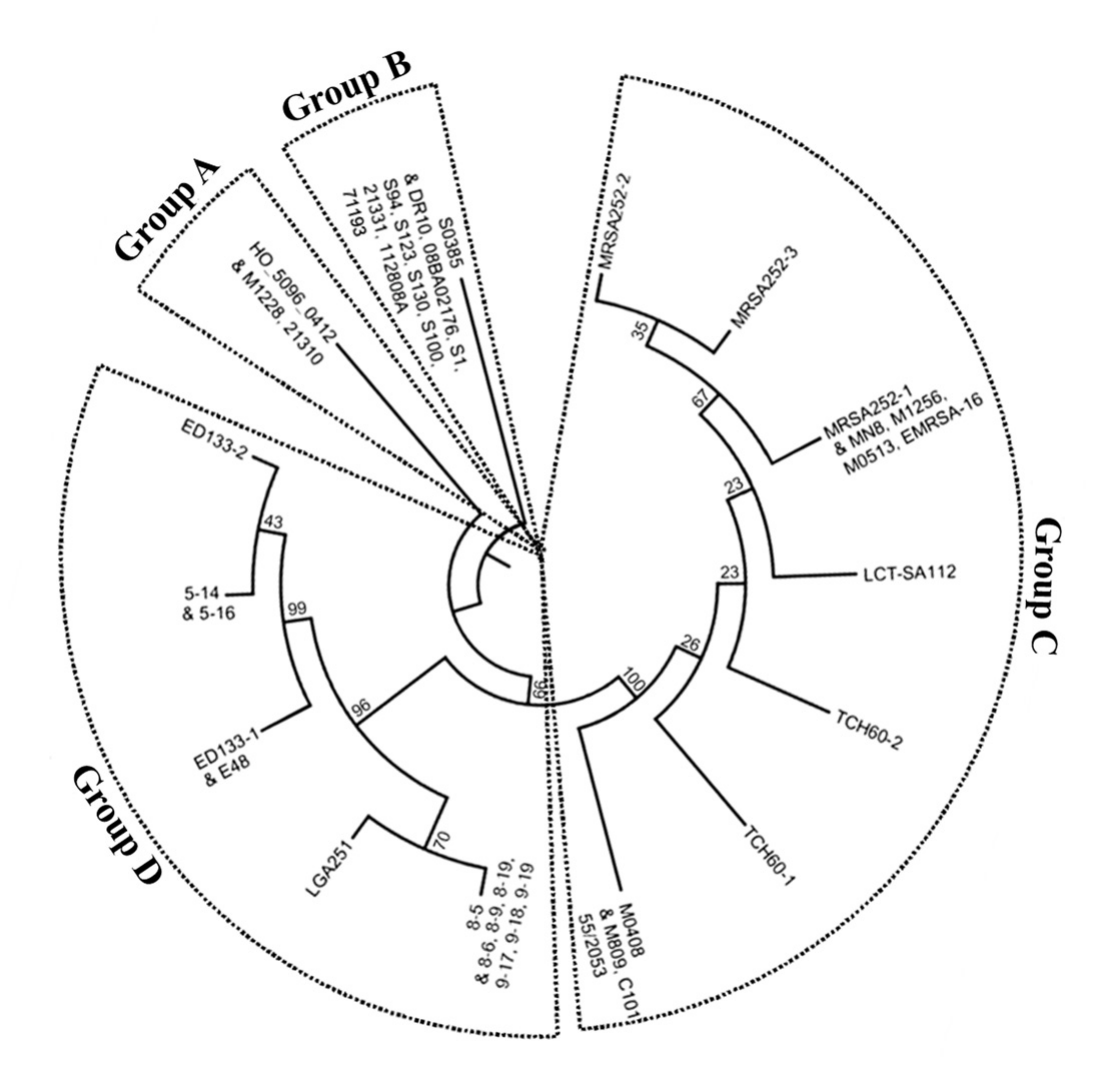
Relationship among the IS*Sau2* containing *S*. *aureus* strains and isolates. The phylogenetic tree of IS*Sau2* gene was constructed by the MEGA software (version 5.2) using default parameters. Numbers at nodes are levels of bootstrap support (percentages) based on a neighbor-joining analysis of 1,000 resampled datasets using the Maximum Composite Likelihood method. Four groups (A, B, C and D) of 27 *S*. *aureus* strains and 10 isolates were indicated. The strains or isolates in a same branch shared identical IS*Sau2* sequences.

In order to determine the diversity of IS*Sau2*, IS*Sau2* PCR products from *S*. *aureus* isolates were sequenced and 13 SNPs were found. Coupled with the BLAST results of IS*Sau2*, a phylogenetic tree was constructed (Fig 1). They were classified into four groups (Groups A, B, C and D) with 14 different IS*Sau2* sequence variants. Each sequence variant was defined as a sequence type. The strains or isolates in an IS*Sau2* sequence type shared identical IS*Sau2* sequences. As shown in Table 2, *S*. *aureus* strains in three groups (Groups A, B and C) all belonged to a same CC- CC398, CC30 and CC22, respectively while Group D consisted of CC133 strains and other singletons. Three strains in Group A were all ST22 isolated from humans. After aligning the amino acid sequences of IS*Sau2* in Group A strains with that in isolate E48 containing a functional transposase, a His to Arg amino acid replacement was identified in the HTH domain, which is responsible for binding of the transposase to DNA. Group B included 11 strains that had an identical IS*Sau2* sequence, IS*Sau2*_1628_, with 32 nucleotides deleted at the 3’ terminus. This mutated sequence missing the IRR could not be recognized by the IS*Sau2* transposase and is therefore presumably inactive in transposition. The strains in this group were isolated from humans or livestock. Group C had 11 strains that display seven IS*Sau2* sequence types, with 3 IS*Sau2* sequence types for MRSA252 and two IS*Sau2* sequence types for TCH60. There were 0–4 SNPs in IS*Sau2* sequences in this group, when M0408 was used as a reference. These strains were all isolated from human infectious diseases. The 12 isolates or strains in Group D were tissue-specific, since they were all isolated from mammary glands with mastitis.

### Insertion positions of IS*Sau2* in IS*Sau2-S*. *aureus*

To define accurate insertion sites of IS*Sau2* in the genomes of *S*. *aureus* isolates, 5’genomic walking was conducted in the genomes of 8 *S*. *aureus* isolates from this study. The PCR products were sequenced. Sequence analysis indicated that IS*Sau2* targets included: a gene encoding a hypothetical phage protein in *S*. *aureus* ED98, a gene encoding a putative membrane protein in *S*. *aureus* LGA251 and a gene encoding a membrane-embedded lipoprotein precursor in *S*. *aureus* RF122 (data not shown).

**Fig 2:**
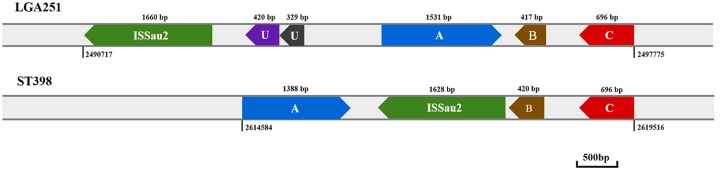
A schematic map of IS*Sau2* and its adjacent sequence in strain LGA251 and strain ST398. The IS*Sau2* and its adjacent sequences were designated as different colors of shapes. The blue “A” represents a hypothetical protein; the brown “B” is a putative membrane protein; the red “C” stands for a putative short chain dehydrogenase; the “U” represents an unknown protein. The length of each region is also indicated.

Our analysis of IS*Sau2* and its adjacent sequences in IS*Sau2*-positive *S*. *aureus* strains also indicated that IS*Sau2*_1628_ in strain ST398 from Group B was closely related to IS*Sau2* of LGA251 in Group D, as shown in Fig 2. In the genome of LGA251, a hypothetical protein containing a hydrolase domain (indicated as A) was located before a putative membrane protein (as B) and a putative short chain dehydrogenase (as C). An IS*Sau2* sequence was found in front of the A/B/C proteins, with a distance of about 2200bp (Fig 2). Similar A/B/C proteins were only found in the genome of strain ST398 and IS*Sau2* was located between protein A and protein B.

## Discussion

This study assessed the presence of IS*Sau2* in 164 *S*. *aureus* isolates from milk samples of mastitic cows in our laboratory and all the *S*. *aureus* strains from the National Center for Biotechnology Information (NCBI) database. Twenty seven *S*. *aureus* strains and 10 isolates were found to contain IS*Sau2* and these isolates and strains could be classified into 4 groups with certain specificities. In particular, those in group D were all isolated from mammary glands therefore exhibiting a strong tissue-specificity. In addition, the isolates used in this study from Shaanxi province in China could be classified in the same group as a Canadian isolate E48 and a UK-sourced strain LGA251. To the best of our knowledge, none of other strains or isolates in the other 3 groups was isolated from mammary glands. Group C strains were all from human sources suggesting host-specificity. Additionally, while Group A strains were also isolated from human infections, group members shared an IS*Sau2* sequence with a mutation in the HTH domain, different from strains in Group C.

It is interesting that the strains in Group B contained a short version of IS*Sau2* (IS*Sau2*_1628_) with the 32bp sequence of its 3’ terminal deleted. In addition, the adjacent genes of IS*Sau2* in strain ST398 of Group B and strain LGA251 were identical (Fig 2). We hypothesize that the relationship between strain ST398 and LGA251 is close. Using the IS*Sau2* in LGA251 as a reference, the IS*Sau2* in strain ST398 is a mutation-rich region with 116 SNPs. *S*. *aureus* CC398 is both a livestock and human pathogen which poses a worldwide threat because of its ability to colonize and infect both humans and animals [21]. CC398 strains lack significant virulence genes and the lineage features a unique genetic background [21]. Because of the specificity of IS*Sau2*_1628_, it could act as a genetic marker for recognizing IS*Sau2*-positive CC398 lineage.

It is widely accepted that transposition must be maintained at a low level due to its detrimental effect on the stability of the host genome [22]. Insertion elements have mechanisms to attenuate their activation. Expression of OrfA/B protein, produced by programmed -1 translational frameshifting, can act as a negative regulator of IS*Sau2* transposition, by competing for DNA binding/catalysis site of OrfAB to indirectly reduce the transposase activity [23]. Moreover, the frequency of frameshifting is also maintained at a low level, approximately 50% in the case of IS*150* [24] and 15% for IS*911* [8]. In addition, the copy number of IS*Sau2* of different *S*. *aureus* strains was low, ranging from 1 to 7 copies. In particular, the copy number of IS*Sau2* in our bovine mastitis-associated *S*. *aureus* isolate was less than three (Table 2). These may contribute to maintaining a low level of transposition of IS*Sau2* in nature.
